# Supplementary material for: Intraspecific diversity in the mechanisms underlying abamectin resistance in a cosmopolitan pest
Source: Evol Appl. 2023 Mar 25;16(4):863–79. doi: 10.1111/eva.13542 (PMC10130554; doi:10.1111/eva.13542)
Supplement: Supplementary file 1 — Figures S1‐S4. [file EVA-16-863-s003.pdf]

SUPPLEMENTARY FIGURES

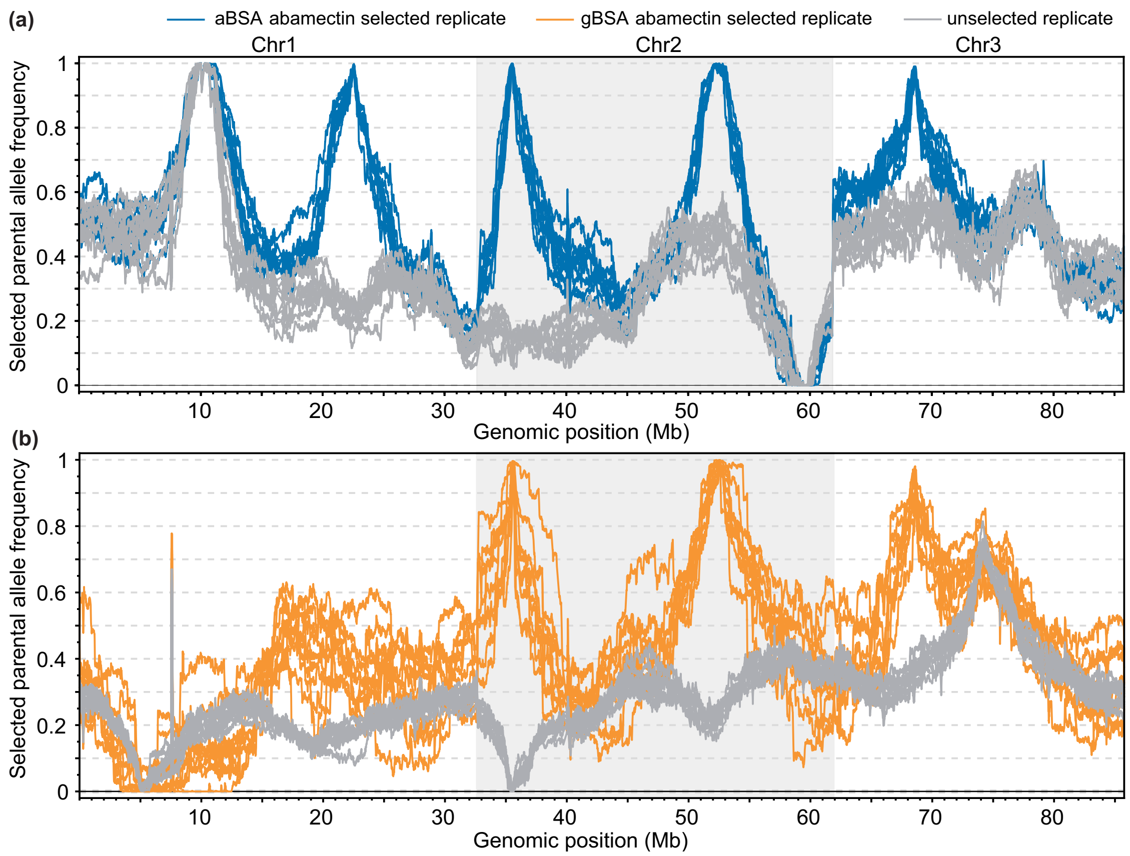

FIGURE S1. Genome-wide allele frequencies (n=10) of the resistant parent in abamectin-selected replicates of aBSA (blue lines) and gBSA (orange lines), and unselected control replicates (grey lines).

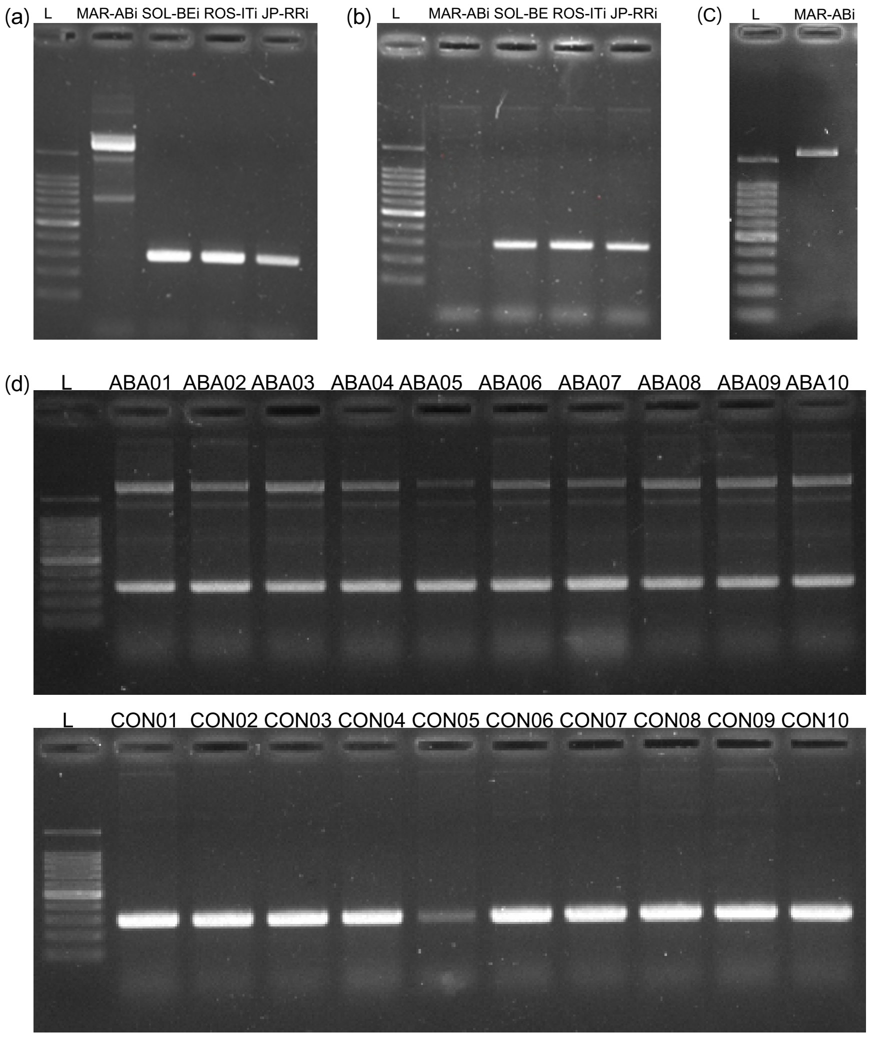

FIGURE S2. 2% agarose gel electrophoresis with PCR amplicons of the fourth exon of *GluCl2*. (a) PCR screening with gDNA from the four parental inbred lines shows a diagnostic 281 bp band for *GluCl2*, and a >1500 bp band when an insertion is present, as in line MAR-ABi. (b) PCR screening with cDNA from the three parental lines SOL-BE, ROS-ITi and JP-RRi shows the diagnostic 281 bp band of *GluCl2*, which is absent in line MAR-ABi. (c) PCR screening of the full-length insertion sequence using gDNA from the MAR-ABi line. (d) PCR screening with gDNA from each abamectin-selected (ABA; top panel) and unselected control replicates (CON; bottom panel) from aBSA. L: 100bp DNA ladder (Promega).

(a)

```
London  GluCl2  TTTGTTTTCGGGGCTTTACTTGAGTTTGCCTTGGTAAACTATGCTTCACGAAGTGATGCT 1080
SOL-BEi GluCl2  TTTGTTTTCGGGGCTTTACTTGAGTTTGCCTTGGTAAACTATGCTTCACGAAGTGATGCT 1080
MAR-ABi GluCl2  TTTGTTTTCGGGGCTTTACTTGAGTTTGCCTTGGTAAACTATGCTTCACGAAGTGATGCT 1080

London  GluCl2  CATCGCCATGCCAGAGAGTTTAATGGTCTAAGGTATCAACGACGATGG-GATAGGGATGG 1139
SOL-BEi GluCl2  CATCGCCATGCCAGAGAGTTTAATGGTCTAAGGTATCAACGACGATGG-GATAGGGATGG 1139
MAR-ABi GluCl2  CATCGCCATGCCAGAGAGTTTAATGGTCTAACCCGGGTAATACTTTTCAGCATTTCATGG 1140
```

inserted site in MAR-ABi

```
London  GluCl2  TAATGTTATCTCGGATGAAACCTCTTATGCACTGAGGCCATTGGTTATCAAAGGAAGTG- 1198
SOL-BEi GluCl2  TAATGTTATCTCGGATGAAACCTCTTATGCACTGAGGCCATTGGTTATCAAAGGAAGTG- 1198
MAR-ABi GluCl2  CAA-----GCTATGACCAAACGAG-CAAGCCTTGCTATAATCTTTTCAAAAAGCAAGCTA 1194

London  GluCl2  ------------------------------------AATACTCAAATAAAAATATATTTT 1222
SOL-BEi GluCl2  ------------------------------------AATACTCAAATAAAAATATATTTT 1222
MAR-ABi GluCl2  GAAACTTTGAACAAATTGTCACAATAAGAACAGGCCAAGACCGTGCTAATATTAACATTA 1254

London  GluCl2  CTCGCTG---GTGGTCAAAGTTTCCA---ACCCGATCTAAACGAATCGATGTTGTGTCGA 1276
SOL-BEi GluCl2  CTCGCTG---GTGGTCAAAGTTTCCA---ACCCGATCTAAACGAATCGATGTTGTGTCGA 1276
MAR-ABi GluCl2  ACCCATGATAATGATCGTACTCATCAGTTACTTGATAAGAAATCATCGGT---------- 1304

London  GluCl2  GAATATTTTTCCCGTTAATGTTTTGCTTATTCAATCTTGTC------TATTGGGTTACTT 1330
SOL-BEi GluCl2  GAATATTTTTCCCGTTAATGTTTTGCTTATTCAATCTTGTC------TATTGGGTTACTT 1330
MAR-ABi GluCl2  ---TAAATTTTCTTGTAATGATACGCTAAACTGTTCTTGGTGAGAAATATTGTTATAATA 1361

London  GluCl2  AT---------------------------------------------------------- 1332
SOL-BEi GluCl2  AT---------------------------------------------------------- 1332
MAR-ABi GluCl2  AGATCACAGTGAGAATGAAGAATACGAAATACGGATCCAAAGAATATTTGCAAAAACAAG 1421

London  GluCl2  ----------------------CTGTT------TCGCCATAAAAGAGATAAAAATGTTTA 1364
SOL-BEi GluCl2  ----------------------CTGTT------TCGCCATAAAAGAGATAAAAATGTTTA 1364
MAR-ABi GluCl2  AATTTGTCATTAATTTGACAATTTATTGCCAAGTTGGCAC---AAAGATGTAAATGATAT 1478

London  GluCl2  TTAA-------------------------------------------------------- 1368
SOL-BEi GluCl2  TTAA-------------------------------------------------------- 1368
MAR-ABi GluCl2  TTCATGGTAAGAATATTGAATTTCAATAGATTGACAAGATATTGAGAATAGAGGAATAAA 1538
```

(b)

```
London  GluCl2  VSLGVTTLLTMATQISGINASLPPVSYIKAIDVWTGVCLAFVFGALLEFALVNYASRSDA 360
SOL-BEi GluCl2  VSLGVTTLLTMATQISGINASLPPVSYIKAIDVWTGVCLAFVFGALLEFALVNYASRSDA 360
MAR-ABi GluCl2  VSLGVTTLLTMATQISGINASLPPVSYIKAIDVWTGVCLAFVFGALLEFALVNYASRSDA 360

London  GluCl2  HRHAREFNGLRYQRRWDRDGNVISDETSYALR----PL---------------VI---K- 397
SOL-BEi GluCl2  HRHAREFNGLRYQRRWDRDGNVISDETSYALR----PL---------------VI---K- 397
MAR-ABi GluCl2  HRHAREFNGLTRVILFSISWQAMTKRASLAIIFSKSKLETLNKLSQ*EQAKTVLILTLTH 419
```

inserted site in MAR-ABi

```
London  GluCl2  -------------GSEYSNKNIFSRWWSKFPTRS-KR-----IDVVSRIFFPLMFC---- 434
SOL-BEi GluCl2  -------------GSEYSNKNIFSRWWSKFPTRS-KR-----IDVVSRIFFPLMFC---- 434
MAR-ABi GluCl2  DNDRTHQLLDKKSSVKFSCNDTLNCSW*EILL**DHSENEEYEIRIQRIFAKTRICH*FD 475

London  GluCl2  --------LFNLVYWVTYLFRHKRDKN-------VY*----------------------- 455
SOL-BEi GluCl2  --------LFNLVYWVTYLFRHKRDKN-------VY*----------------------- 455
MAR-ABi GluCl2  NLLPSWHKDVNDISW*EY*ISID*QDIENRGINRKYEIN*IRKLFFPGIFDYF*LIGQDL 530

London  GluCl2  ------------------------------------------------------------ 455
SOL-BEi GluCl2  ------------------------------------------------------------ 455
MAR-ABi GluCl2  GMVFNYPCWLMDGHGLGR*GGTQKNQQQENCEKLNLMNAVMMQQRTCQVLMALIRLTVMK 589

London  GluCl2  -------- 455
SOL-BEi GluCl2  -------- 455
MAR-ABi GluCl2  RLVLSIHH 597
```

FIGURE S3. *GluCl2* gene from several *Tetranychus urticae* lines. Alignments of a portion of *GluCl2* using (a) nucleotide sequences and (b) amino acid sequences from abamectin - susceptible and -resistant parental lines (SOL-BEi and MAR-ABi) and the reference strain London. Red arrows indicate the start of the insertion in line MAR-ABi, which causes a premature stop. Sequences shared between the three lines have a black background (>90% similarity), while sequences shared between two lines have a grey background.

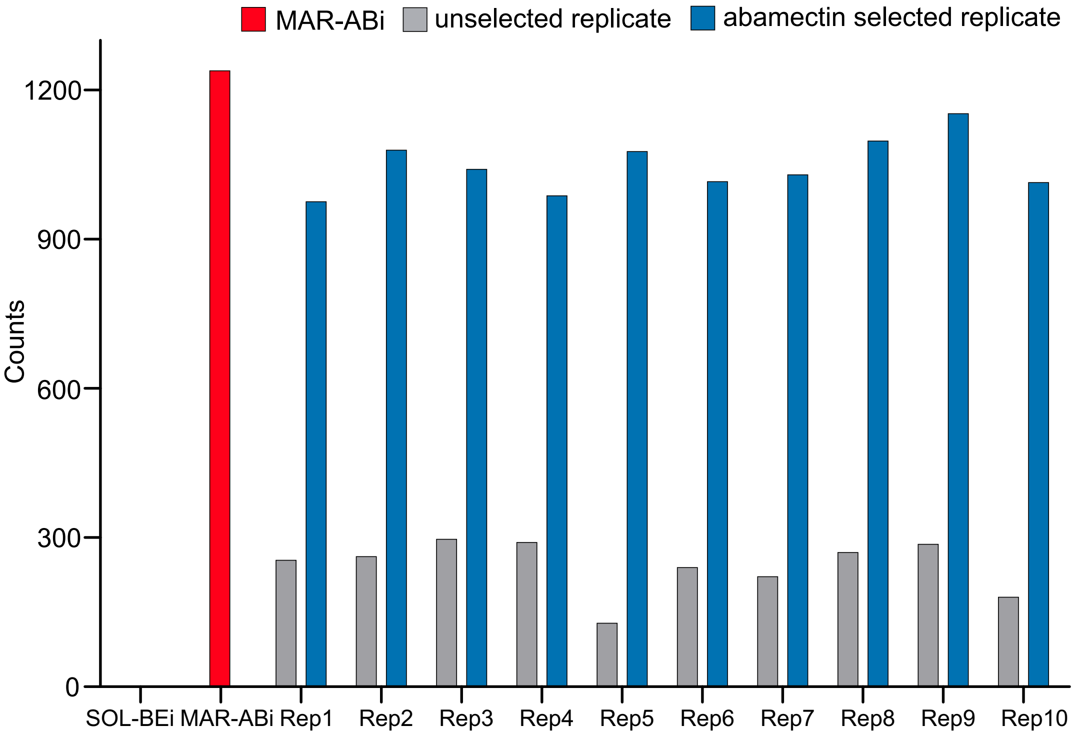

FIGURE S4. Number of DNA reads from the genomes of the parental lines (MAR-ABi and SOL-BEi) and the experimental populations of aBSA (abamectin-selected and unselected replicates) that mapped to the insertion sequence in *GluCl2*.

SUPPLEMENTARY TABLES

Table S1. Differentially expressed genes (Log2 Fold Change (Log2FC) > |2|, padj <0.05) in the comparison of the resistant parent MAR-ABi and the susceptible parent SOL-BEi of experiment aBSA

Table S2. Differentially expressed genes (Log2 Fold Change (Log2FC) > |2|, padj <0.05) in the comparison of the resistant parent ROS-ITi and the susceptible parent JP-RRi of experiment gBSA

Table S3. Genes within a 500kb bracket around the top genomic window of QTL peak 1 in aBSA, located at ~22.5Mb on Chr1

Table S4. Overlapping genes within a 500kb bracket around the top genomic window of QTL peak 2 in aBSA and gBSA, located at ~2.8Mb on Chr2

Table S5. Genes within a 500kb bracket around the top genomic window of QTL peak 3 in aBSA, located at ~19.4Mb on Chr2

Table S6. Genes within a 500kb bracket around the top genomic window of QTL peak 3 in gBSA, located at ~19.9Mb on Chr2

Table S7. Overlapping genes within a 500kb bracket around the top genomic window of QTL peak 4 in aBSA and gBSA, located at ~6.6Mb on Chr3

Table S8: Allele frequency of resistance mutations in *TuGluCl1* and *TuGluCl3* in all replicates and parental strains of aBSA and gBSA
