# Supplementary material for: Intraspecific diversity in the mechanisms underlying abamectin resistance in a cosmopolitan pest
Source: Evol Appl. 2023 Mar 25;16(4):863–79. doi: 10.1111/eva.13542 (PMC10130554; doi:10.1111/eva.13542)
Supplement: Supplementary file 2 — Supplementary file 1 [file EVA-16-863-s001.docx]

Supplementary file 1

>TuGluCl2_insertion sequence

ACCCGGGTAATACTTTTCAGCATTTCATGGCAAGCTATGACCAAACGAGCAAGCCTTGCTATAATCTTTTCAAAAAGCAAGCTAGAAACTTTGAACAAATTGTCACAATAAGAACAGGCCAAGACCGTGCTAATATTAACATTAACCCATGATAATGATCGTACTCATCAGTTACTTGATAAGAAATCATCGGTTAAATTTTCTTGTAATGATACGCTAAACTGTTCTTGGTGAGAAATATTGTTATAATAAGATCACAGTGAGAATGAAGAATACGAAATACGGATCCAAAGAATATTTGCAAAAACAAGAATTTGTCATTAATTTGACAATTTATTGCCAAGTTGGCACAAAGATGTAAATGATATTTCATGGTAAGAATATTGAATTTCAATAGATTGACAAGATATTGAGAATAGAGGAATAAATCGTAAATATGAGATCAATTGAATTAGAAAATTGTTTTTTCCTGGAATTTTTGACTATTTTTGACTAATTGGGCAAGATCTTGGAATGGTCTTTAATTATCCTTGCTGGTTGATGGACGGTCACGGTCTTGGCAGGTAGGGAGGGACGCAAAAAAACCAGCAGCAAGAAAACTGCGAGAAATTAAACCTGATGAACGCAGTCATGATGCAACAAAGAACTTGCCAAGTTCTCATGGCTCTAATTCGTCTAACAGTCATGAAAAGCCTGGTTTTGTCGATTCATCACCAAAAAATGATTTGTCTGAACCGAATCAGGATGATATTGACAGTGATGTAACGATATCAGGATCTCATGAAGAAAATCAGGAACAAGGAGATTCTTCACCGATCAATCTTCAGTCATTTAAAGTAAATCAAGTTGGCGACCTAAATGATAATCATTTTGAAGACGAAGAAACAATTGAATGATCTTGGCAATTTCTTGGCAAAATATTACTTATTATTTTTTGATACTTTTGAGTTTATATAGATTAAATTATTAAGAAATCTTTCAATATTTCAATAAAATTTTTTTGCAAATAATGACAAGATCTTATTTAGTTGTTGCCAAGAAATCAATCGTGAATCAGTGCAAAATCATGAAAAGATTGTGCACAAGAATACATCATGATTTTTAGCGAAAAATTAGCTGTTTCTTGCTGGTATTTCCTTTCATTTCTTGTGATGATTCGATTTTGTCTTTTGCCAAGGAAACAGTGAAGTCGTATCTATTTCTTTTTGAATTTTTCTGGTAAGATCAAACACTTATTAGTAGTGTTCCAGAAAATTTCTGACAATTTTTTGCCATGAAAGCATAAGAGAATCGACCGAAATTTTATCTTCTTAACTTGCTAATTCTTCAGTAATGCTGTGCCCTTCAAGAATCTTATATGACTTAATATGATCATATCAATAAAATATGCAGATAAATAGTCTGAACATGAGTAAAAAATTGTTAATGCATAACAAAAGCTTGATGGAAATACTCGACATTAAAATGTGCCAAGCGATTTGCTATATTCTTGTCGAAAAGTTCTTGCCCATTTTCATGCTATGACTGCCTGAAAGTATTACCCGGGA
